# Promoting mental health in Portuguese college students - A pilot study with Yoga-based relaxation techniques

**DOI:** 10.1192/j.eurpsy.2025.1998

**Published:** 2025-08-26

**Authors:** A. Ilharco, M. Pocinho, M. J. Brito, A. P. Amaral

**Affiliations:** 1 Unidade Local de Saúde de Coimbra; 2 Escola Superior de Tecnologia da Saúde de Coimbra, Instituto Politécnico de Coimbra; 3 Serviço de Psiquiatria, Unidade Local de Saúde de Coimbra; 4 Instituto de Psicologia Médica, Faculdade de Medicina da Universidade de Coimbra, Coimbra; 5 Centro de Investigação e Inovação em Educação (inED), Politécnico do Porto, Porto, Portugal

## Abstract

**Introduction:**

Mental health of higher education students is a relevant topic, especially for students in the health field. The development of relaxation techniques in this population is increasingly recognized as crucial to promote psychological well-being and reduce distress.

**Objectives:**

1. Study the associations between mental health and lifestyle in Portuguese college students in the health field; 2) Evaluate the effectiveness of a Yoga-based relaxation techniques on mental health and physiologic variables.

**Methods:**

Study 1 had a cross-sectional design, with 107 participants, 76,6% females, average age of 21,33 years (SD=4,88).Study 2 employed a pretest-posttest design, with 3 timepoints: T0, T1, T2, with T2 being a follow-up after 3 months. Participants: 11 students, 81,8% females, average age of 26,82 years (SD=11,59).

Measures: 1) Mental Health Inventory (MHI-38); 2) Lifestyle questionnaire. In study 2, the intervention consisted of six face-to-face sessions, lasting 60 minutes each, with practical training of Yoga-based relaxation techniques and evaluation of heart and respiratory rate, and blood pressure before and after each session.

**Results:**

Study 1 – 32,7% presented Distress, and we found significative correlations between sleep quality perception, global health perception and Mental Health (*p*<.001); male students who practice physical activity have more Positive Affect. Study 2 – In all sessions, the physiologic variables decrease in T1, and Distress and Anxiety decrease significantly in T1 (*p*=0,037; *p*=0,031, respectively). After the follow-up, the improvement in mental health remains significative (*p*<0,001).

**Conclusions:**

The results suggest a relevant percentage of students with distress, and that the intervention program with Yoga-based relaxation techniques contribute to decrease heart, respiratory and the blood pressure rate. After the intervention and the follow-up, there is a decrease in distress in Portuguese college students. Suggestions for future studies would be to replicate the intervention with a control group and a larger sample.

**Disclosure of Interest:**

None Declared

